# Changes in Fat-Free Mass, Protein Intake and Habitual Physical Activity Following Roux-en-Y Gastric Bypass Surgery: A Prospective Study

**DOI:** 10.1007/s11695-023-06650-y

**Published:** 2023-05-30

**Authors:** Malou A. H. Nuijten, Thijs M. H. Eijsvogels, Boy Sanders, Laura M. Vriese, Valerie M. Monpellier, Eric J. Hazebroek, Ignace M. C. Janssen, Maria T. E. Hopman

**Affiliations:** 1 Department of Medical BioSciences (Route 928), Radboud University Medical Center, P.O. Box 1901, 6500 HB Nijmegen, The Netherlands; 2 Nederlandse Obesitas Kliniek, Huis Ter Heide, The Netherlands; 3 Department of Surgery, Rijnstate Hospital/Vitalys Clinics, Arnhem, The Netherlands

**Keywords:** Roux-en-Y gastric bypass surgery, Fat-free mass, Protein intake, Physical activity

## Abstract

**Purpose:**

Large inter-individual variations in post-bariatric fat-free mass loss (FFML) are observed, which might relate to differences in protein intake and physical activity across patients. We performed repetitive assessments of protein intake and physical activity before and after banded Roux-en-Y gastric bypass surgery, and examined its relations to FFML during 6 months of follow-up.

**Materials and Methods:**

FFML (bio-impedance analyses), protein intake (24-h dietary recalls) and moderate-to-vigorous physical activity (MVPA; activPAL) were assessed in 28 patients (4 males, age 42 ± 12 years) before surgery and at 1-, 3- and 6-months post-surgery. Changes in protein intake and MVPA were evaluated with mixed model analysis, whereas associations with FFML were assessed by univariate regression analysis.

**Results:**

Six-month FFML was -7.3 ± 3.6 kg. Protein intake decreased from 80 ± 29 g/day (pre-surgery) to 45 ± 26 g/day (1 month post-surgery (*P* < 0.001)) and did not improve thereafter (51 ± 21 g/day; *P* > 0.05). Seven participants (25%) consumed ≥ 60 g protein/day at 6 months post-surgery. Participants performed 7394 ± 2420 steps/day in 54 ± 20 min/day of MVPA, which did not change from pre- to post-surgery (*P* > 0.05). A higher step count (B = -0.002; 95%CI = [-0.004 – 0.000]; *P* = 0.048) and higher level of MVPA (B = -0.29; 95%CI = [-0.54 – -0.03]; *P* = 0.018) were related to a lower FFML.

**Conclusion:**

A lower post-surgery FFML was attributable to higher MVPA levels but not protein intake. This may be due to the low total protein intake and the observation that only a minority of patients achieved a protein intake ≥ 60 g/day. Future studies should focus on interventions to increase post-bariatric protein intake and MVPA levels.

**Graphical Abstract:**

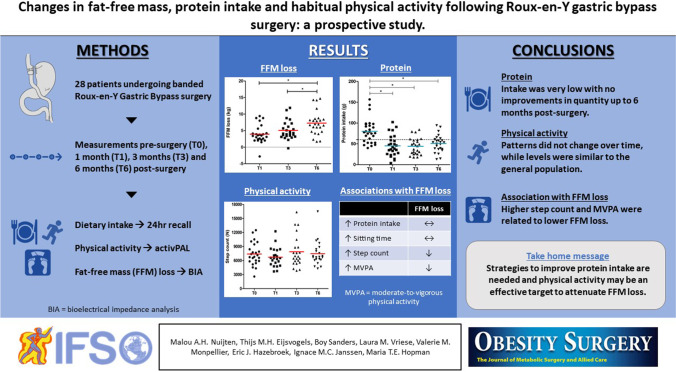

**Supplementary Information:**

The online version contains supplementary material available at 10.1007/s11695-023-06650-y.

## Introduction

Bariatric surgery is considered the most effective strategy to achieve substantial and prolonged weight loss in individuals with severe obesity [1, 2]. Unfortunately, bariatric surgery is also associated with high rates of fat-free mass (FFM) loss, of which skeletal muscle tissue is the main component [3]. FFM is essential for maintaining functional capacity and metabolic health, as well as potentially preventing an adaptive hunger response after weight loss [4–6]. The magnitude of FFM loss may therefore be related to higher mortality and health care expenditures [7, 8]. Therefore, it is important to limit FFM loss in conjunction with maximizing weight loss within the bariatric population.

Recent studies show a large interindividual variation in post-bariatric FFM loss (FFML), which cannot fully be explained by preoperative patient characteristics [9]. It is therefore likely that postoperative behavior, such as diet and physical activity, play an important role in FFML. Sufficient dietary protein intake is of great importance for FFM preservation [10], and since protein cannot be stored in the body, adequate daily protein intake is essential. Post-bariatric guidelines state that a minimum of 60 g protein per day should be consumed, up to 1.5 g/kg ideal bodyweight on an individualized basis [11]. Furthermore, habitual physical activity is a known stimulus for muscle growth by increasing muscle protein synthesis rates [12] and is therefore positively associated with skeletal muscle mass [13]. On the other hand, the lack of the stimulus for muscle growth in periods of disuse or restricted physical activity, is known to induce rapid loss of muscle mass [14].

Thus, adequate protein intake and physical activity are essential to maintain muscle mass. Previous studies assessed the impact of exercise [15], protein [16–18] and combined interventions [19, 20] after bariatric surgery, but the effects on FFM are inconclusive and high levels of drop outs and low adherence were observed. Further insights into physical activity and diet during the first postoperative months (i.e. the window with ~ 78% of total FFML) in relation to FFML are therefore warranted [3]. We aimed to obtain detailed insight into protein intake and physical activity levels up to 6 months after bariatric surgery, and to examine its relations to FFM loss. We expected a decrease in daily protein intake and an increase in physical activity levels from pre- to 6 months post-surgery, and hypothesized that higher levels of protein intake and physical activity were related to a lower FFML. With more detailed knowledge on both factors within this early-postoperative time frame, we can identify targets to retain FFM during perioperative care.

## Materials and Methods

### Study Population

Participants were enrolled via the *Nederlandse Obesitas Kliniek*, a healthcare organization that provides an interdisciplinary care program for patients undergoing bariatric surgery; consisting of pre- and post-bariatric group counseling focused on lifestyle change [21]. Due to COVID-19 restrictions, participants could not be included on-site, but were invited for this study via e-mail. Eligible participants were invited if they were scheduled for a primary banded Roux-en-Y gastric bypass (RYGB) procedure (Minimizer Gastric Ring, width = 7.0 cm (females) or 7.5 cm (males)) and participated in the perioperative care program. Exclusion criteria were (i) secondary or revisional procedures, (ii) non-obesity related co-morbidities that affect muscle tissue (e.g., muscle diseases), (iii) inability to walk (e.g., wheelchair bound) and (iv) inability to understand and perform the scheduled procedures. After response, interested patients were informed and screened via telephone and included upon their first day of measurements. This study was conducted in accordance with the Declaration of Helsinki, ethical approval was obtained via METC Oost-Nederland [#2019–5731] and all participants provided informed consent prior to assessments.

### Perioperative Care Program

The perioperative lifestyle program focuses on adopting a healthy lifestyle and strives for self-reliance of the patients. The program is provided by an interdisciplinary team consisting of a physician, dietician, psychologist, physiotherapist, internist and surgeon. Patients are enrolled in groups of ten individuals.

#### Preoperative Program

During the 6-week preoperative program, patients participate in group visits on a weekly basis. The group visits consist of three consecutive 1-h sessions with a psychologist, dietician and physiotherapist. The goal of this phase is to educate patients about healthy eating habits and physical activity behavior, and to encourage intrinsic motivation towards lifestyle changes. Furthermore, the clinic holds a strict policy for mandatory weight loss (i.e., -3.5 kg when BMI < 55 kg/m^2^ and -10 kg when BMI ≥ 55 kg/m^2^) in order to increase operation safety and to prove motivation for lifestyle changes. Therefore, the preoperative program contains two diet-phases: the preparation diet and the crash diet. Neither of the diets focuses on caloric limitations to prevent obsessive behavior of counting calories. The preparation diet starts upon the first preoperative session and focuses on regular food intake (5–6 meals/day), sufficient fluid intake (1.5 L/day) and food intake with low content of fat and sugars. The crash diet starts four weeks prior to surgery and focuses on decreasing portion sizes, practicing new eating techniques and getting used to a new eating pattern. Usual waiting times from start of the program up to operation date are between 42 to 84 days.

#### Postoperative Program

After surgery, patients continue the group visits up to 12 months post-surgery (13 sessions). In addition, patients have a medical consultation (including weight measurements) at 1, 3, 6, 9, 12, and 18 months post-surgery. The goal of this stage is to become self-reliant in their new healthy lifestyle. During the program, patients are not subjected to a specific diet or physical activity regimen, but are educated and counseled regarding their physical activity behavior and eating habits. In addition, patients are coached on SMART (Specific, Measurable, Assignable, Realistic and Time-related) goal setting and were trained to recognize and cope with body signals like pain, exhaustion and fatigue [21]. The postoperative diet starts with 4–5 days of fluid diet, in which we advise to consume protein-rich fluids (e.g. milk). After five days, patients are allowed to consume solid food products. Dietary advice focuses on regular food intake, eating techniques and food product choices and discusses the importance of protein intake. This advice includes consumption of a protein-rich product at each meal (including snacks) and consumption of 2 to 3 dairy products per day, preferably high in proteins (i.e., > 7 g protein per 100 ml with < 5 g sugars). Assessment of protein intake is no standard of care and protein supplementation is merely recommended in exceptional cases with severe complications or low tolerability towards general food intake.

### Measurements

In this prospective cohort study, we assessed body composition, dietary intake and physical activity at 4 time points: prior to the surgical procedure (T0), and at 1 month (T1), 3 months (T3) and 6 months after surgery (T6).

#### Body Composition

Height was measured using a non-elastic measuring tape. Body weight, FFM and fat mass were determined by bioelectrical impedance analysis (BIA; Tanita BC-420MA) at all time points. The BIA method is considered useful to repeatedly assess body composition in clinical settings [22]. Body mass index (BMI), percentage of total weight loss (%TWL) and proportion of FFM loss within total weight loss (%FFML/WL) were calculated [23].

#### Dietary Intake

Daily dietary intake was determined by two 24-h recalls per timepoint, which was previously defined as a valid representation of protein intake [24]. All recalls were performed by trained interviewers to ensure a more representative food assessment compared to self-administered recall methods [25] and to make the assessment eligible for low-income and low-literacy individuals. The assigned days were randomized over the week, with the restriction that no participant was assigned to two identical weekdays (e.g., Monday and Monday), two weekend days (e.g., Saturday and Sunday) or two consecutive days (e.g., Tuesday and Wednesday). The first recall was performed face-to-face during the visit, whereas the second recall was performed by phone. During the recall, participants reported all food and beverages that they consumed the day before in detail regarding quantity and brand. Portion sizes could be documented in frequently used household items, which were subsequently quantified with standard portion sizes. All recalls were performed and coded by trained research assistants and entered into the program *Compleat* [26]. Energy and macronutrient intake was then calculated, using the Dutch food composition table (NEVO, 2013). The mean of the two recalls represented the daily dietary intake. Outcomes were expressed in absolute intake (kcal and g) and relative intake (en%).

#### Physical Activity

Physical activity pattern was objectively measured with a waterproofed activity-monitor (ActivPAL minor), that was validated to accurately assess sedentary time, stepping activity and activity intensity [27, 28]. The monitor consists of a small device (25 × 45 × 5mm), which is attached to the upper thigh by the research assistant upon their visit and continuously worn for 8 consecutive days. A self-reported sleep schedule in combination with a modified version of a previously developed algorithm was used to identify sitting, standing and stepping during wear-time [29]. The algorithm also checked validity of each measurement day. A measurement day is considered invalid when (i) one activity takes up more than 95% of total awake time, (ii) step count is below 1000 or (iii) number of awake hours is less than 10. A valid measurement consists of ≥ 5 valid days, including at least 1 weekend day. Furthermore, the algorithm can distinguish moderate-to-vigorous physical activity (MVPA) from light physical activity (LPA), based on time spent stepping with metabolic equivalent of task values ≥ 3 (i.e., an intensity of activities with an energy expenditure ≥ 3 times greater than resting metabolism). MVPA is therefore continuously measured (with an accuracy of 1 to 5 s) and no minimal effort or bout is needed to be considered MVPA. Outcomes are sitting time (h/day), active time (h/day), step count, time spent in LPA (min/day) and time spent in MVPA (min/day). Twelve ActivPAL measurements were missing due to devices’ error (*n* = 4), allergic reaction to tape (*n* = 2), invalid measurements (*n* = 3) and lost in the mail (*n* = 5).

### Statistical Analysis

Statistical analyses were performed using SPSS 27 software (IBM SPSS Statistics for Windows, Version 27 IBM Corp., Armonk, NY, USA). All continuous variables were visually inspected and tested for normality with the Shapiro-Wilk test. Continuous parametric data were displayed as mean ± standard deviation and categorical data as count (percentage). Time dependent changes in body composition-, dietary intake- and physical activity parameters were assessed by linear mixed model analysis (repeated covariance type = AR1), with the measuring timepoints as fixed factor. In case of an overall significant time-effect, pairwise comparisons with a Bonferroni correction were performed to compare the main effects between timepoints. Furthermore, the postoperative averages over time (T1, T3 and T6) were calculated for protein intake (g/day), sitting time, step count, LPA and MVPA. Univariate linear regression analysis was performed with 6-month %FFML/WL or FFML (kg) as dependent factor and mean protein intake, sitting time, step count, LPA and MVPA as independent factors. Statistical significance was assumed at *P* < 0.05 (two-sided).

## Results

### Study Population

A total of 34 participants were included in this study, but five participants withdrew within 1 month post-surgery. Participants were included for analysis if they had at least two body composition measurements and (i) either physical activity and dietary intake on two timepoints, or (ii) physical activity or dietary intake on three timepoints (Supplemental Fig. 1). This resulted in a population of 28 participants (4 males and 24 females) with a mean age of 42 ± 12 years [range = 21 – 65 years]. Baseline characteristics of included participants were comparable to the excluded participants (Supplemental Table 1). Due to COVID-19 restrictions many surgical procedures were postponed, which resulted in a range in preoperative measurements from 12 to 175 days before surgery (mean = 77 ± 48 days). None of the participating subjects used anti-obesity medication.

#### Body Composition

**Fig. 1:**
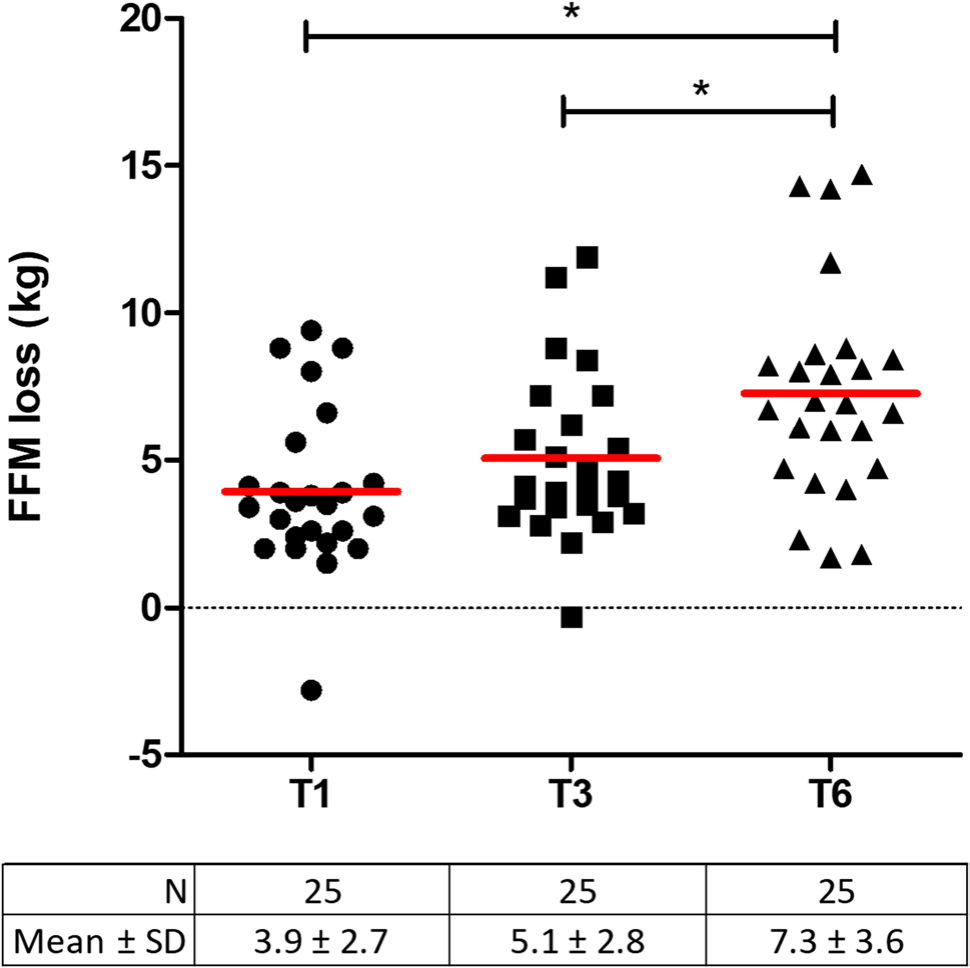
Interindividual variation in fat-free mass loss over time. Each point reflects an individual participant and red lines represents the mean loss at the particular time point. The participant that showed an increase in FFM at T1 and T3 was a male (65 years-old) with a weight loss of 29.9 kg (T6), a high preoperative protein intake (156.7 g/day), a high step count at T0 and T1 (both > 12,000 steps/day), and a relatively high protein intake at T1 (68.5 g/day). FFM = fat-free mass, T1 = 1 month post-surgery, T3 = 3 months post-surgery and T6 = 6 months post-surgery

Participants lost 32.8 ± 6.9 kg bodyweight and 7.3 ± 3.4 kg FFM over 6 months, reflecting 22.7 ± 12.1%FFML/WL (Table 1). Large interindividual differences in body composition changes up to T6 were observed [ranges: 21.7 to 45.0 kg WL; 1.7 to 14.7 kg FFM and 6.0 to 53.9% FFML/WL] (Fig. 1).

**Table 1:** Pre- to postoperative changes in body composition parameters

	T0 (*n* = 28)	T1 (*n* = 25)	T3 (*n* = 25)	T6 (*n* = 25)	Time effect (*P*-value)
Weight (kg)	121.0 ± 16.8	108.0 ± 16.3^a,b^	97.5 ± 14.9^a,b^	89.6 ± 13.4^a,b^	< .001
BMI (kg/m^2^)	43.2 ± 6.5	38.6 ± 6.5^a,b^	34.9 ± 6.0^a,b^	32.0 ± 5.4^a,b^	< .001
Fat percentage (%)	49.6 ± 5.1	46.6 ± 6.1^a,b^	42.2 ± 7.1^a,b^	39.0 ± 8.5^a,b^	< .001
FM (kg)	60.4 ± 12.7	50.8 ± 12.8^a,b^	41.7 ± 12.3^a,b^	35.6 ± 12.0^a,b^	< .001
FFM (kg)	60.6 ± 8.3	57.2 ± 7.7^a,b^	55.8 ± 7.3^a^	54.0 ± 7.1^a,b^	< .001
TWL (%)	NA	11.4 ± 3.1	19.5 ± 3.7^b^	26.8 ± 4.0^b^	< .001
FFML/WL (%)	NA	30.0 ± 24.5	21.8 ± 13.0^b^	22.7 ± 12.1	< .001

Parametric continuous variables are displayed as mean ± SD

*BMI* body mass index, *FM* fat mass, *FFM* fat-free mass, *FFML/WL* percentage of fat-free mass loss within weight loss, *TWL* total weight loss, *NA* not applicable, *T0* preoperative measurement, *T1* 1 month post-surgery, *T3* 3 months post-surgery, *T6* 6 months post-surgery

^a^Significantly different from baseline; ^b^ Significantly different from previous timepoint

#### Dietary Intake

**Fig. 2:**
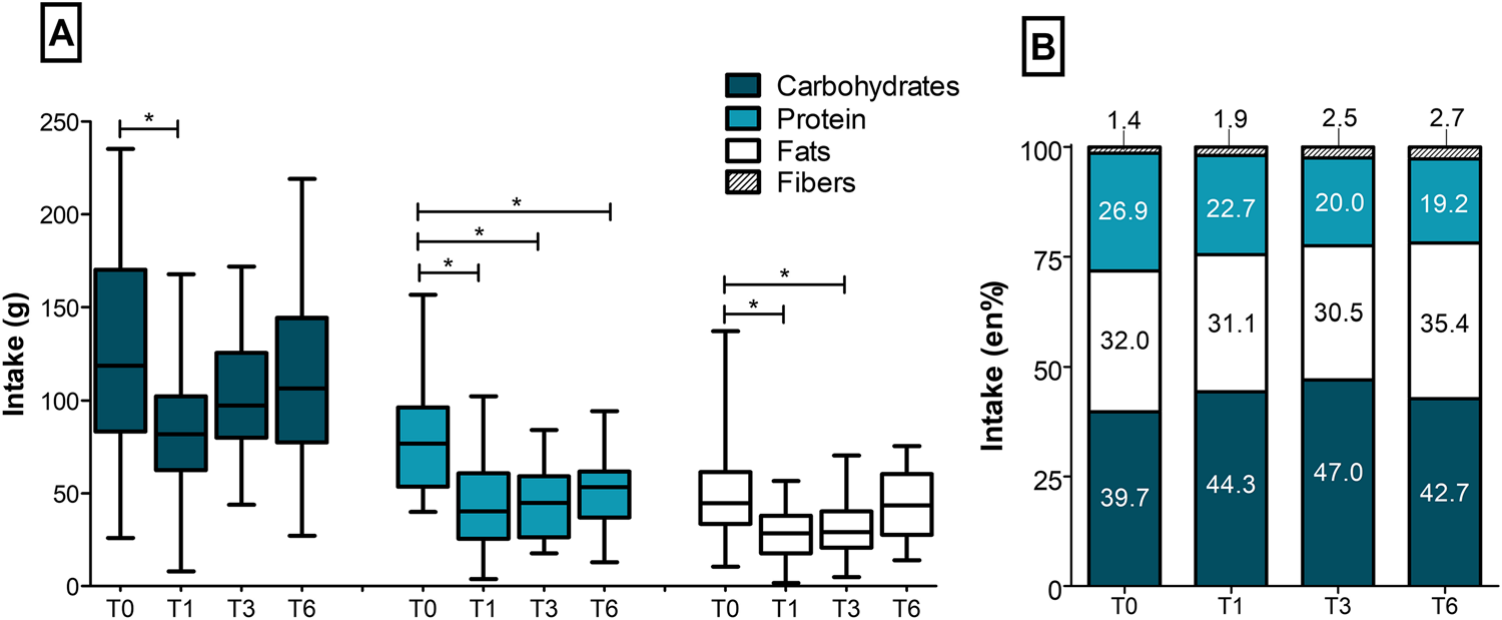
Dietary intake of macronutrients over time in grams per day (**A**) and energy percentage (**B**) at preoperative measurements (T0), 1 month post-surgery (T1), 3 months post-surgery (T3) and 6 months post-surgery (T6). *P < 0.05

Energy intake drastically decreased from pre-surgery to 1 month post-surgery, and then gradually increased up to 6 months post-surgery (T0: 1311 ± 491 kcal; T1: 797 ± 348 kcal; T3: 899 ± 335 kcal; and T6: 1077 ± 395 kcal). Intake of all macronutrients followed a similar pattern over time, with a significant decrease in intake from T0 to T1, but no significant changes between T1, T3 and T6 (Fig. 2A). Relative energy intake (en%) shifted towards relatively higher fat- and carbohydrate intake and a lower protein intake over time compared to preoperative measures (T0 vs. T3; *P* = 0.002 and T0 vs. T6; *P* < 0.001; Fig. 2B).

**Fig. 3:**
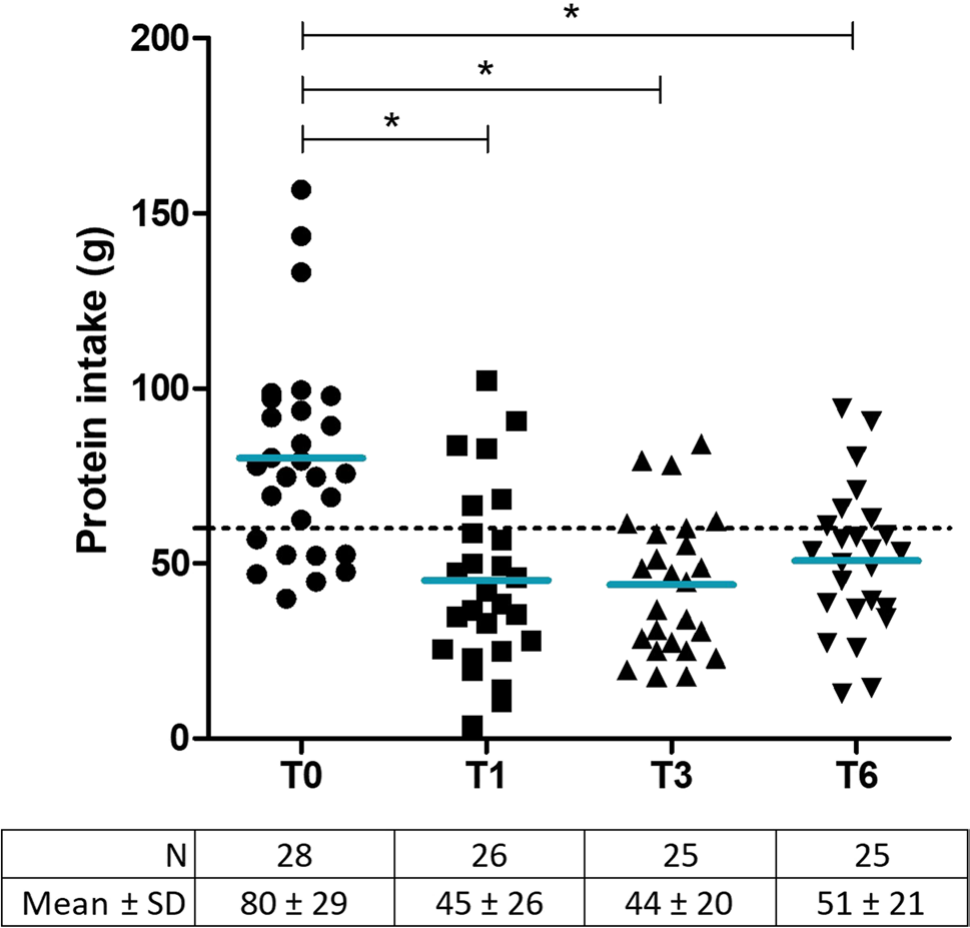
Protein intake over time in grams per day. Each dot reflects one participant and blue lines represent the mean intake. The dashed line represents the minimal protein recommendation of 60 g/day. T0 = preoperative measurement, T1 = 1 month post-surgery, T3 = 3 months post-surgery and T6 = 6 months post-surgery. *P < 0.05

**Fig. 4:**
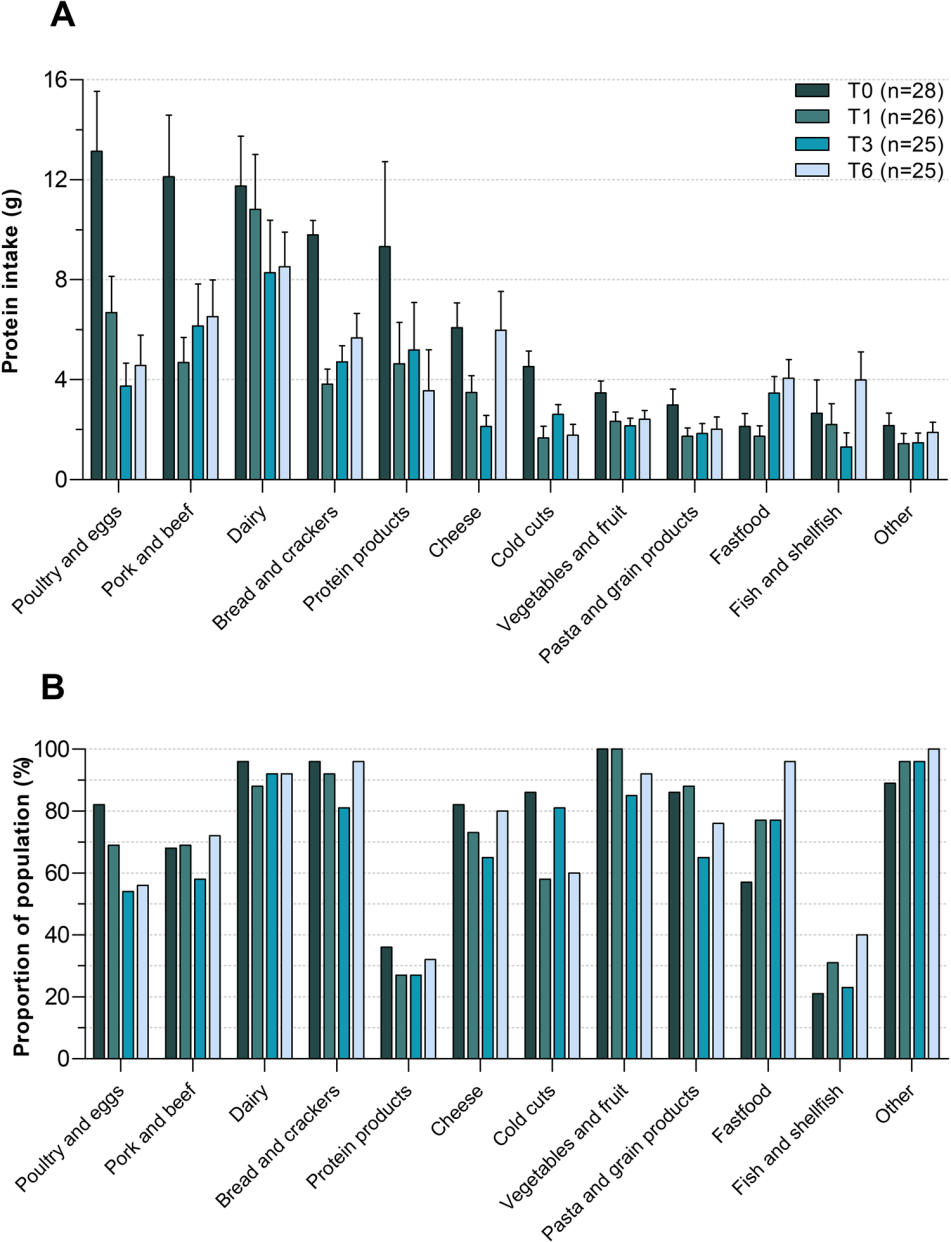
Mean and standard error of dietary protein intake from different food categories (**A**) at pre-surgery (T0; *n* = 28), 1 month (T1; *n* = 26), 3 months (T3; *n* = 26) and 6 months (T6; *n* = 25) post-surgery. Percentage of participants consuming from the food categories at each timepoint (**B**)

Postoperative protein intake was significantly lower at 1 month post-surgery (45.2 ± 25.5 g/day, Fig. 3) compared to pre-surgery (80 ± 29 g/day), and did not change at 3 and 6 months of follow-up. Hence, only seven participants (28%) consumed over ≥ 60 g protein per day at 6 months post-surgery. Participants that consumed ≥ 60 g protein/day were significantly older (39 ± 13 vs. 50 ± 9 years; *P* = 0.048), whereas other body characteristics were similar between groups (Supplemental Table 2). Preoperatively, protein was mostly derived from poultry and eggs, followed by pork, beef and dairy products (Fig. 4). The amount of protein intake derived from poultry, pork and beef and the proportion of patients consuming these products substantially declined postoperatively, whereas dairy products became the most predominant source of protein. Furthermore, protein products such as protein bars and protein yoghurts were consumed by 27% (T1 and T3) and 32% (T6) of the participants.

#### Physical Activity

**Fig. 5:**
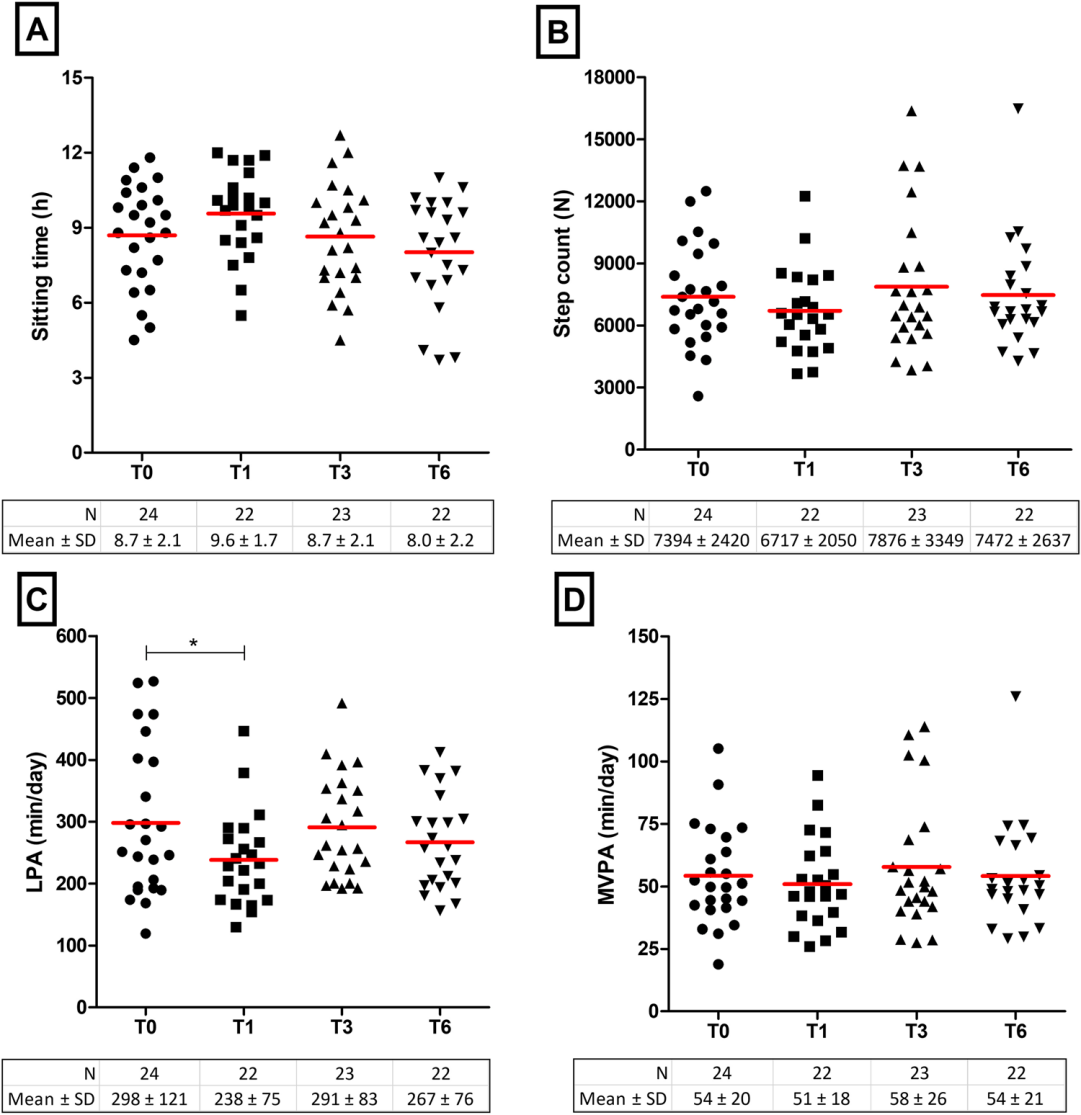
Changes in physical activity over time for sitting time (**A**), step count (**B**), light physical activity (**C**) and moderate-to-vigorous physical activity (**D**). Each dot reflects one participant and red lines represent the group average. T0 = preoperative measurement, T1 = 1 month post-surgery, T3 = 3 months post-surgery and T6 = 6 months post-surgery

Before surgery, participants spent 298 ± 121 min/day in LPA, 54 ± 20 min/day in MVPA, with a daily sitting time of 8.7 ± 2.1 h and a step count of 7394 ± 2420. Daily minutes in LPA were significantly lower at 1 month post-surgery compared to preoperative LPA levels, but normalized at 3- and 6 months post-surgery. Sitting time, step count and MVPA did not change over time (Fig. 5).

#### Fat-Free Mass Loss, Protein and Physical Activity

Univariate linear regression analysis showed that a higher step count and MVPA level were related to lower %FFML/WL (B = -0.002, 95%CI = [-0.004–0.000], *P* = 0.048 and B = -0.29, 95%CI = [-0.54–-0.03], *P* = 0.031, respectively). Similar results were found for FFML **(**Table 2**)**. No associations with protein intake, sitting time or LPA were found for either %FFML/WL or FFML.

**Table 2:** Univariate linear regression between mean protein intake and physical activity parameters (T1 – T3 – T6) with 6-month %FFML/WL and FFML (kg)

		%FFML/WL			FFML (kg)		
		Beta	95%CI	*P*-value	Beta	95%CI	*P*-value
Mean (T1-T3-T6)	Protein (g)	-0.02	[-0.29 – 0.24]	0.85	-0.02	[-0.09 – 0.06]	0.62
	Sitting time (h)	0.52	[-3.04 – 4.07]	0.77	-0.04	[-1.08 – 1.01]	0.95
	Step count (N)	-0.002	[-0.004 – 0.000]	0.048	-0.001	[-0.001 – 0.000]	0.024
	LPA (min/day)	0.006	[-0.08 – 0.09]	0.88	0.001	[-0.02 – 0.02]	0.98
	MVPA (min/day)	-0.29	[-0.54 – -0.03]	0.031	-0.09	[-0.17 – -0.02]	0.018

Univariate linear regression analysis was performed for 6-month %FFML/WL and FFML(kg) with protein intake and all physical activity parameters as independent variables

*%FFML/WL* proportion of fat-free mass loss within weight loss, *FFML* fat-free mass loss (6 months), *LPA* light physical activity, *MVPA* moderate-to-vigorous physical activity, *Beta* beta coefficient, *95%CI* 95% confidence interval, *T1* 1 month post-surgery, *T3* 3 months post-surgery, *T6* 6 months post-surgery

## Discussion

This study aimed to obtain detailed insight into the associations of lifestyle changes (i.e., dietary patterns and habitual physical activity) and FFML following bariatric surgery. We report the following main findings: 1) patients had 7.3 ± 3.6 kg of FFML with a large interindividual variation (1.7 to 14.7 kg FFML), 2) postoperative protein intake was poor, with only seven patients (25%) reaching a protein intake over 60 g/day at 6 months post-surgery, 3) patients were more physically active than expected (7472 ± 2637 steps/day) and physical activity parameters did not change over time and 4) a higher daily step count and time spent MVPA were related to lower %FFML/WL and FFML, whereas no association with protein intake was found. These findings indicate that post-bariatric care programs should actively target protein intake and stimulate habitual physical activity in order to retain FFM in conjunction with maximal weight loss.

Perioperative care programs stimulate healthier food choices, including a sufficient intake of high-quality protein [30] due to its role in muscle metabolism, wound healing and weight control [31, 32]. We observed a poor protein intake of 45 ± 25 g/day at 1 month post-surgery, which marginally improved over time. Importantly, only 25% of our participants met the minimal daily protein intake recommendation of 60 g/day. The low protein intake is probably attributable to a combination of factors. First, the bariatric procedure induces restriction and thereby drastically decreases the overall food intake. Second, protein is associated with an increased satiety [33], which may further limit energy consumption. Furthermore, alterations in taste perception and olfactory changes can predispose an aversion to certain protein-rich foods, such as meat and poultry [34]. Also, poor chewing techniques and reduced digestion can cause difficulty in processing solid high-protein products, possibly causing more gastrointestinal complaints upon ingestion [35, 36]. This may cause patients to substitute products with high biological value for foods that are easier to digest (i.e., with a lower biological value and a higher carbohydrate content) [35]. For example, dairy seemed to be well tolerated and became the predominant source of protein after surgery. Our observations align with data from previous studies [37–39] and highlight an important ‘window of opportunity’ to further optimize post-bariatric care. Taken together, post-surgery protein intake is insufficient in the large majority of bariatric patients, so targeted intake strategies are needed, which may focus on dairy and high-protein products.

Increases in physical activity are related to better weight maintenance, improved health outcomes [40, 41] and a better regulation of appetite and food intake [42]. Therefore, physical activity is an important target for improving bariatric outcomes. Our study population was more physically active than expected, with activity levels similar to the general population [43]. A potential explanation for our relatively high activity levels compared to cohorts from other countries may relate to differences in cultural norms and infrastructure (e.g., another Dutch cohort found similar ranges in step count [44]). Another possibility is that physically active patients were more inclined to engage in this study than less active patients because the study corresponded with their general interests. Still, our findings align with some [45] but not all previous studies among bariatric patients as also higher [44, 46] and lower [47, 48] activity levels were reported. Nonetheless, no changes in sitting time, step count, LPA or MVPA were found in our cohort, suggesting that the population remains moderately active within the first 6 months post-surgery.

Our %FFML/WL was in line with other post-RYGB studies [3, 49], but large ranges were observed [6.0 to 53.9% FFML/WL]. Preoperative patient characteristics were previously found to explain little (~ 5%) of the inter-individual variation in FFML that is typically observed following bariatric surgery [9]. In this study, we found that postoperative step count and MVPA levels were inversely related to FFML, meaning that participants with higher activity levels showed less FFML. These findings are contradictory to some [50, 51] but not all previous studies [52–54], and may relate to differences in sample sizes, follow-up duration or measurement tools. As association analysis could not disentangle whether exercise promotes FFM preservation or vice versa, future prospective randomized clinical trials are needed to confirm the power of exercise to attenuate FFML.

In contrast to our hypothesis, no association between postoperative protein intake and FFML was found. A potential explanation for this neutral finding may relate to the low levels of protein intake that were present in our population results. Indeed, previous studies that did find a positive association between protein intake and muscle mass retention had larger numbers of patients with protein intake levels over 60 g/day [50, 51]. Hence, sufficient protein intake should be a key target for post-bariatric care programs, in order to maintain FFM as much as possible. Regular monitoring of dietary intake in the initial postoperative months is therefore recommended. Patients with a low protein intake can either (i) increase protein consumption with well-tolerated products (e.g., dairy), (ii) use protein-enriched products (e.g., high-protein yoghurts), or (iii) start with protein supplementation. To enhance compliance of such strategies, future studies should examine tolerability of various quantities (g/day) and protein quality (i.e., protein source) after surgery, in order to establish feasible protocols for protein intake tailored to the bariatric population.

A limitation of this study was that preoperative measures may not reflect normal preoperative behavior, since patients were enrolled in a lifestyle program and the study took place during COVID-19 restrictions. These restrictions should be considered in interpreting the findings and future studies may further clarify these aspects. Still, the impact on study outcomes was limited since analyses focused on postoperative physical activity and protein intake in relation to FFML. Another limitation was the use of BIA to assess FFM since this method assumes a constant hydration status and body fluid distribution, which may be affected in individuals with obesity [55]. Hereby, previous studies reported overestimation [56] and underestimation of FFM in individuals with obesity [57]. But more importantly, BIA appeared to have a repeatable and constant bias when the same machine and measurement protocol is used in a longitudinal design [57]. This suggests that the risk of bias induced by BIA is limited in our study, since we use repetitive measurements with the same device. Furthermore, caution regarding interpretation of protein intake with respect to guidelines is warranted since evidence for the current protein guidelines is low (grade D) and more research is needed to determine how much protein is needed after bariatric surgery. Finally, since banded RYGB may yield different results compared to non-banded RYGB [58], the translation of our findings to other bariatric procedures should be further examined.

In conclusion, postoperative protein intake was very low and did not improve up to 6 months post-surgery, with only 25% of participants reaching protein recommendations. No association between protein intake and FFML was found, possibly caused by the generally low levels of protein. Postoperative care should therefore actively focus on strategies to improve protein intake. Physical activity parameters were similar to activity levels of the general population and did not change over time following surgery. Furthermore, participants with higher daily step count and MVPA levels showed less FFML, suggesting that physical activity may be an effective target to improve FFM retention.

## Supplementary Information

Below is the link to the electronic supplementary material.

Supplementary Figure 1 Flowchart of study population and missing values before surgery (T0), and at 1 month (T1), 3 months (T3) and 6 months post-surgery (T6). BC = body composition, DI = dietary intake, PA = physical activity. (JPG 154 KB)

Supplementary file2 (DOCX 28 KB)

## Data Availability

The data underlying this article were provided by Nederlandse Obesitas Kliniek (NOK) by permission. Data could be shared upon request to the corresponding author and following approvement by the NOK. These third-party data are not freely available, and cannot be shared publicly because of contractual restriction.
