# Supplementary figures and images for: Changes in Fat-Free Mass, Protein Intake and Habitual Physical Activity Following Roux-en-Y Gastric Bypass Surgery: A Prospective Study
Source: Obes Surg. 2023 May 30;33(7):2148–57. doi: 10.1007/s11695-023-06650-y (PMC10228447; doi:10.1007/s11695-023-06650-y)

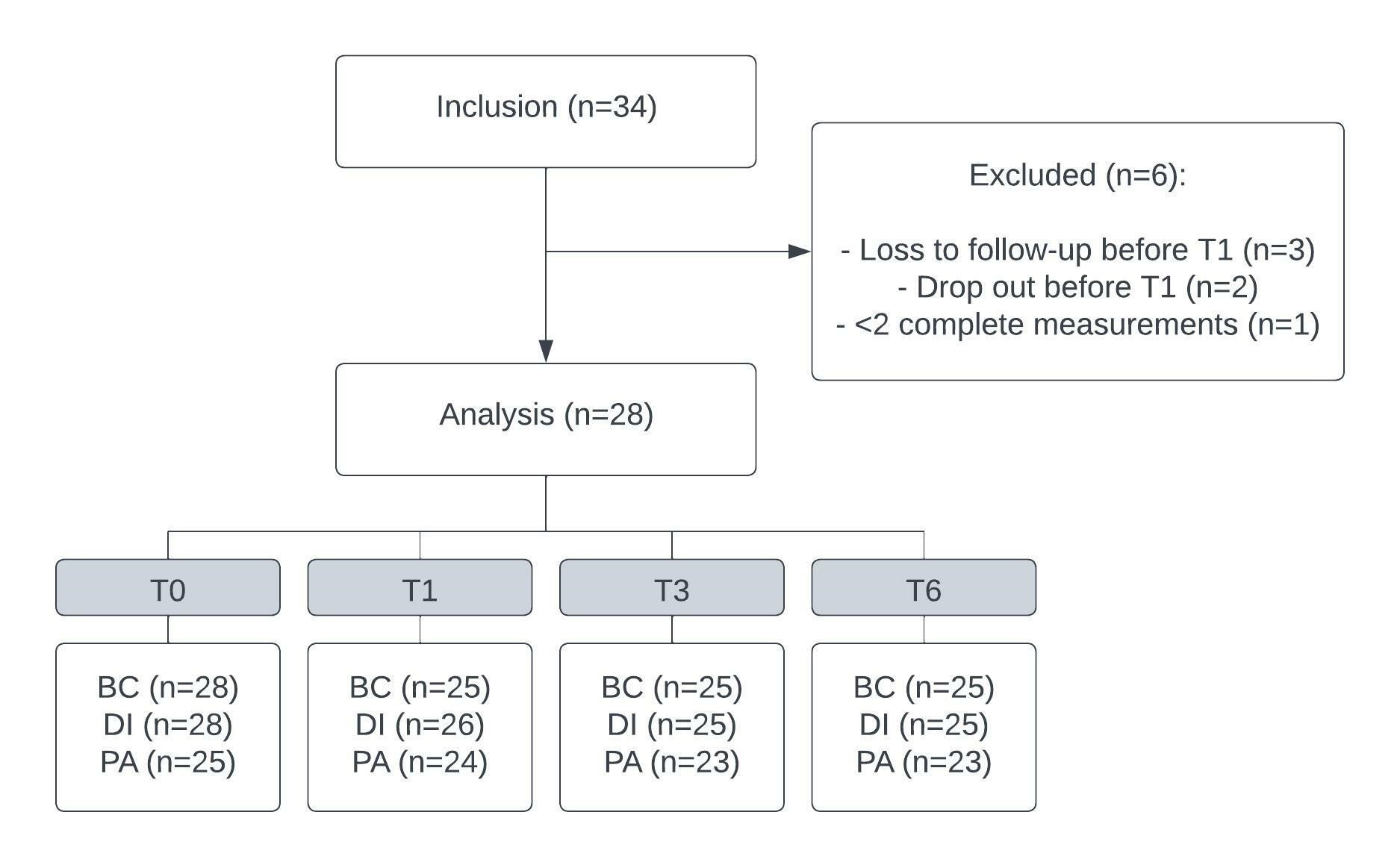

Supplement: Supplementary file 1 — Supplementary Figure 1 Flowchart of study population and missing values before surgery (T0), and at 1 month (T1), 3 months (T3) and 6 months post-surgery (T6). BC = body composition, DI = dietary intake, PA = physical activity. (JPG 154 KB) [file 11695_2023_6650_MOESM1_ESM.jpg]
