# Supplementary material for: Changes in Fat-Free Mass, Protein Intake and Habitual Physical Activity Following Roux-en-Y Gastric Bypass Surgery: A Prospective Study
Source: Obes Surg. 2023 May 30;33(7):2148–57. doi: 10.1007/s11695-023-06650-y (PMC10228447; doi:10.1007/s11695-023-06650-y)
Supplement: Supplementary file 2 — Supplementary file2 (DOCX 28 KB) [file 11695_2023_6650_MOESM2_ESM.docx]

| **Supplemental Table 1.** Baseline characteristics of included vs. excluded participants | | | |
| --- | --- | --- | --- |
|  | **Included (n=28)** | **Excluded (n=6)** | **P-value** |
| Age (years) | 42 ± 12 | 35 ± 10 | 0.18 |
| Body weight (kg) | 121.0 ± 16.8 | 124.9 ± 35.0 | 0.68 |
| BMI (kg/m^2^) | 43.2 ± 6.5 | 44.8 ± 12.8 | 0.65 |
| Fat percentage (%) | 49.6 ± 5.1 | 50.0 ± 4.4 | 0.87 |
| FM (kg) | 60.4 ± 12.7 | 63.1 ± 21.0 | 0.68 |
| FFM (kg) | 60.6 ± 8.3 | 61.9 ± 14.7 | 0.78 |
| Continuous, parametric variables are displayed as mean ± SD. Differences between groups were tested by an independent t test. BMI = body mass index, FM = fat mass, FFM = fat-free mass. | | | |

| **Supplemental Table 2.** Characteristics of participants with a protein intake <60 g/day versus ≥60 g/day. | | | |
| --- | --- | --- | --- |
|  | **Protein intake** | |  |
|  | **<60 g/day** (n=18) | **≥60 g/day** (n=7) | **P-value** |
| Age, *years* | 39.3 ± 12.5 | 50.1 ± 8.9 | 0.048 |
| Males, *N(%)* | 2 (11%) | 2 (29%) | 0.29 |
| Length, *m* | 1.67 ± 0.07 | 1.70 ± 0.07 | 0.22 |
| Weight, *kg* | 122 ± 19 | 123 ± 13 | 0.89 |
| BMI, *kg/m^2^* | 43.9 ± 7.6 | 42.4 ± 4.1 | 0.64 |
| FFM, *kg* | 60 ± 8 | 65 ± 9 | 0.11 |
| Fat mass, *kg* | 62 ± 15 | 58 ± 8 | 0.42 |
| Fat percentage, *%* | 51 ± 5 | 47 ± 4 | 0.10 |
| Weight loss, *kg* | 32.5 ± 7.1 | 33.5 ± 6.7 | 0.76 |
| TWL, *%* | 26.6 ± 4.0 | 27.1 ± 4.2 | 0.80 |
| FFML, *kg* | 7.1 ± 2.9 | 7.7 ± 5.1 | 0.70 |
| FFML/WL, *%* | 22.1 ± 9.8 | 24.2 ± 17.5 | 0.71 |
| Continuous variables are displayed as mean ± standard deviation, whereas categorical variables are displayed as count (percentage). Differences between groups were assessed by independent t-test for continuous variables and Chi-Square test for categorical variables.  BMI = body mass index; FFM = fat-free mass; TWL = total weight loss; FFML = fat-free mass loss; FFML/WL = proportion of fat-free mass loss within weight loss. | | | |
